# Urine Metabolite Profiles after the Consumption of a Low- and a High-Digestible Protein Meal, and Comparison of Urine Normalization Techniques

**DOI:** 10.3390/metabo14040177

**Published:** 2024-03-22

**Authors:** Nadezda Khodorova, Juliane Calvez, Serge Pilard, Simon Benoit, Claire Gaudichon, Douglas N. Rutledge

**Affiliations:** 1 UMR Physiologie de la Nutrition et du Comportement Alimentaire, Université Paris-Saclay, AgroParisTech, INRAE, UMR PNCA, 91120 Palaiseau, France; juliane.calvez@agroparistech.fr (J.C.); simon.benoit@inrae.fr (S.B.); claire.gaudichon@agroparistech.fr (C.G.); 2 Plateforme Analytique, Université de Picardie Jules Verne, 80000 Amiens, France; serge.pilard@u-picardie.fr; 3 Faculté de Pharmacie, Université Paris-Saclay, 91400 Orsay, France; douglas.rutledge@agroparistech.fr; 4 Muséum National d’Histoire Naturelle, 75005 Paris, France

**Keywords:** animal protein, ANOVA-based factorial method, chemometrics, human nutrition, plant protein, urinary metabolomics

## Abstract

In the context of dietary transition toward plant proteins, it is necessary to ensure protein security in populations. It would thus be of interest to identify biomarkers of altered protein digestibility in populations. We examined the association between urinary metabolites and the acute intake of low- or highly digestible protein in healthy volunteers. The urine samples were collected before and 9 h after the ingestion of a meal containing either no protein, zein (low-digestible) or whey protein isolate (highly digestible). The liquid chromatography–high resolution mass spectrometry metabolomics approach was used for the profiling of the urinary metabolites. For the standardization of metabolomics data sets, osmolality-based, standard normal variates (SNV) and probabilistic quotient normalization (PQN) techniques were used. The ANOVA-based factorial method, AComDim_ICA, was used for chemometrics analysis. The osmolality adjustment has a beneficial effect and the subsequent mathematical normalization improves the chemometric analysis further. Some changes in the urinary metabolomes were observed 9 h after the meal in the three groups. However, there was no difference in the urine metabolome between groups. No biomarker of protein digestibility can be identified after the ingestion of a single meal, even when marked differences in the digestion efficiency of protein have been observed.

## 1. Introduction

Diets with reduced intake of animal products are gaining popularity for ethical, environmental and health-related reasons. The switch between protein sources is one of the key aspects of this transition, with recommendations for lower intake of animal protein (AP). However, plant and animal proteins have distinct amino acid (AA) compositions and when excluding protein from animal food sources, the risk of consuming a nutritionally inadequate diet increases. 

In recent years, metabolomics has been introduced into epidemiologic research in the field of nutritional sciences. Several studies aimed to identify the metabolite patterns between omnivores, vegetarians and vegans. The distinct metabolite profiles are reported between meat eaters and vegans. The plasma of vegans shows lower total cholesterol levels and acylcarnitines, and indispensable AA (IAA) pools are also lower than for omnivores [1,2,3,4]. In urine, significantly different patterns of metabolites were also reported between vegetarians and omnivores. The urine of meat consumers contained higher concentrations of creatinine, glycine, mannitol, urea and phosphocholine [5,6]. Even if most of these metabolites reflect differences in consumption of animal and plant proteins (PP), APs and PPs are not consumed alone but as whole foods and the results obtained are influenced by the dietary environment. Only one study has investigated the differences in the plasma metabolome specifically associated with AP and PP intake [7,8]. Thus, knowledge about the link between metabolomics signature and dietary protein quality is still limited. 

APs and PPs display different AA compositions, but PPs also have a lower digestibility than APs. In humans, it has been shown that mean AA digestibility values were higher than 90% for meat, milk or eggs, whereas they ranged from 60 to 80% for plant-based proteins such as maize proteins or whole legumes [9]. The non-digested dietary nitrogen and AAs are excreted in feces while the absorbed AAs are metabolized and their metabolites can be excreted in urine. Hence, urine metabolite profiles may reflect a strong contrast in digestibility. Indeed, in a previous study of urinary metabolome changes in rats after consuming beef, we could distinguish specific metabolites, depending on the cooking process, that affected protein digestibility [10].

Hence, this work aimed to investigate if urine metabolites can be used to discriminate between individuals after an acute intake of low-digestible plant protein and highly digestible animal protein. The urinary metabolome of a previous clinical study was analyzed. In this study, we compared whey protein isolate (WPI) and zein ileal digestibility, the latter being among the lowest, i.e., 60%, of all protein foods tested in humans [11]. The secondary aim was to evaluate the pre- and post-acquisition methods of urine normalization for the standardization of metabolomics data sets. In our work, we compared a biological normalization technique (pre-acquisition dilution of urine to a uniform osmolality value), mathematical techniques (post-acquisition methods applied to the data such as standard normal variates (SNV)) and probabilistic quotient normalization (PQN). In order to evaluate the effect of the type of protein consumed, the difference in composition of the urine samples before and after the meal, and the interaction of these two factors, a recently developed ANOVA-based factorial method, AComDim_ICA, was used.

## 2. Materials and Methods

### 2.1. Subjects and Test Meals

The study has been described in detail in our previous publication [11]. Before entering the study, all volunteers provided written consent for their participation. Exclusion criteria included subjects with a body mass index (BMI; in kg/m^2^) <18 or >30, allergies to latex, cow milk proteins or maize, positive serology for HIV and hepatitis B and C viruses, pregnancy, severe chronic disease and abusive drug or alcohol consumption. The study was approved by the Ethical Committee of Sud Méditerranée III (ref. number 2017.05.01 bis) and was registered at www.clinicaltrials.gov as NCT03279211 https://www.clinicaltrials.gov/study/NCT03279211?id=NCT03279211&rank=1 (3 June 2022). A total of 22 volunteers were included (WPI, *n* = 7; zein, *n* = 8; protein-free, *n* = 7).

The test meal consisted of a 400 g test drink, containing either 29 g of zein (Zein, 89.0% proteins; Sigma-Aldrich, Saint Quentin Fallavier, France), 29 g of WPI (Whey Protein Isolate 894, 90.6% proteins; Fonterra, Auckland, New Zealand) or no protein (protein-free group) in a mixed-fruit smoothie (Innocent, Paris, France), and 120 g of protein-free biscuits. The meal with no protein was used in the initial study to evaluate endogenous losses of nitrogen and AA and determine the true ileal nitrogen and AA digestibility of the test proteins [11], but in the present study, the protein-free group was used as a control. The subjects ingested the test meal on the morning of the experiment, after overnight fasting. During the following 9 h, they only drank 200 mL of water hourly. Urine samples were collected before and then 9 h after the ingestion of the test meal.

### 2.2. Urine Sample Preparation and LC-MS Analysis

The urine samples which were not normalized for osmolality were diluted 1:10 (batch 1) with milliQ water/acetonitrile (90/10, *v*/*v*) and filtered using 0.2 nm Phenex-RC Syringe Filters with a cellulose membrane (Phenomenex, Le Pecq, France). For osmolality normalization, urine samples were first diluted with Milli-Q-grade water to the lowest specific osmolality (measured by Roebling micro osmometer) and then diluted 1:10 with milliQ water/acetonitrile (batch 2). Quality control (QC) samples were prepared by combining small aliquots of all samples for each batch separately. 

An Acquity H-Class system was coupled to a Q-TOF Synapt G2 Si instrument (Waters Corporation, Milford, MA, USA). Analytes were separated using two chromatographic techniques that permit detection of either apolar or polar compounds: reverse-phase chromatography (RP) and hydrophilic interaction chromatography (HILIC). The RP chromatography was performed on an Acquity CSH C18 column (2.1 × 100 mm; 1.7 µm bead size; Waters). Column temperature was 40 °C and the eluents A and B were 0.01% formic acid in water and 0.01% formic acid in acetonitrile, respectively. The gradient was run at 0.4 mL/min and consisted of an isocratic elution for 0.5 min of 5% B, and then B was increased at a linear rate to 95% in 10 min, then re-equilibrated for 1 min with 5% B and held at 5% B until 16 min. For HILIC, an Acquity BEH Amide column (2.1 × 100 mm; 1.7 µm bead size; Waters) was used. Column temperature was 45 °C and the eluents were acetonitrile:20 mM of pH 3.5 ammonium formate (50:50, *v*:*v*; eluent A) and acetonitrile:20 mM of pH 3.5 ammonium formate (90:10, *v*:*v*; eluent B). The HILIC gradient was run at 0.6 mL/min and consisted of an isocratic elution for 2.5 min of 100% B, then B decreased at a linear rate to 0% in 10 min, then re-equilibrated for 1 min with 100% B and held at 100% B until 15 min. The injection volume was 1 µL for both RP and HILIC.

Electrospray ionization was performed in positive electrospray ionization mode (ESI+) with the following settings: capillary voltage at 3.0 kV; cone voltage at 20 V; source offset 20 V, mass range from 50 to 1100 Da. The TOF was operated in the resolution mode, providing an average resolving power of 25,000 (FWHM) and leucine-enkephalin was used as a lock mass to correct mass accuracy. The MS spectra were recorded in the centroid mode at 0.2 sec/scan. Samples were injected in random order together with blanks and QCs. For blank samples, milliQ water/acetonitrile (90/10, *v*/*v*) was used.

### 2.3. Chemometrical Methods 

#### 2.3.1. LC-MS Data Processing and Matrix Pretreatment

Features were extracted from the chromatograms using MarkerLynx (MassLynx V4.1, Waters) with the following parameters: intensity threshold, 500 counts; extracted ion chromatogram (EIC) window, 0.03 Da; signal-to-noise threshold, 8; and retention time window, 0.2 min. Isotope peaks were removed by MarkerLynx. 

The 4 data sets (RP and HILIC, with and without osmolality adjustment), consisting of blanks, QCs and samples obtained before and 9 h after ingestion of the 3 types of protein meals (WPI, zein, protein-free), were all imported into Matlab version 7.6.0 (Mathworks Inc., Natick, MA, USA) for clean-up prior to chemometric analysis. The first clean-up step consisted in removing all variables with an intensity in the blanks greater than 1/100 their mean intensity in the other samples. The next step was to remove from all the samples any variables that were not present in the QCs.

#### 2.3.2. Normalization Methods

Normalized data sets were created by applying in-house Matlab functions SNV [12,13] and PQN [14] to the signals of all the data sets. These two methods are the most commonly used mathematical pretreatments for the correction of global variations in signal intensity. The SNV method aims to reduce differences in the global intensities of the signals resulting from dilution effects, while the PQN method determines a theoretical dilution factor for each sample by using the distribution of the quotients calculated as the ratios of the intensity of each of its variables over that of the corresponding variable in a reference signal. An Independent Components Discriminant Analysis (IC-DA) [15] was applied to each data set to examine the effect of SNV and PQN on the dispersion of the IC-DA Scores and to evaluate the quality of the separation of the groups of samples. 

#### 2.3.3. Data Analysis

In order to determine which experimental factors and interactions have a significant effect on the results, an approach based on the ANOVA paradigm was applied to separate variations into main effects, interactions and noise by creating a series of matrices that are calculated as the means of the variables at each level of each factor in an experimental design, and which are successively subtracted from the original matrix to obtain the final matrix of residual errors. These matrices are then used to evaluate the significance of each of these effects compared to the residual error. 

In the case of ANOVA-PCA (APCA) [16] and ANOVA-SCA (ASCA) [17], each of these matrices is analyzed separately by Principal Components Analysis and Simultaneous Components Analysis, respectively. 

ANOVA-Common Dimensions (AComDim) [17], on the other hand, offers the possibility to have an overall picture of all the sources of variation by means of a single model. As with APCA and ASCA, one starts with a single experimental matrix, out of which a set of factor and interaction matrices are generated. All the data tables calculated by adding the residuals matrix back to each factor matrix are modeled simultaneously with the multi-table method ComDim (or Common Components and Specific Weights Analysis—CCSWA) [18]. The replacement of the PCA step at the core of the ComDim algorithm by Independent Components Analysis (ICA) yields the so-called ComDim-ICA and once the set of factor and interaction matrices and the residual matrix has been built from X, one can apply ComDim-ICA instead of the classical ComDim, resulting in the so-called AComDim-ICA method [19]. To verify that a separation of the factor levels could not have been obtained with random factor data, a permutation test was performed. The samples were given 199 random group attributions and then submitted to AComDim-ICA. The F-values for the permuted samples were calculated along with the sum of the correlations between the original and the permuted group attributions. If the influence of the factor is real, the F-values should increase with the correlation.

The most significant variables for the separation of the factor levels were determined using an S-plot based on the correlations and covariances calculated between the Global Scores calculated by AComDim-ICA and each X matrix variable. A *t*-test was then applied to compare the intensities of the variables selected by the S-plot in the different data sets.

## 3. Results 

### 3.1. Normalization

**Figure 1:**
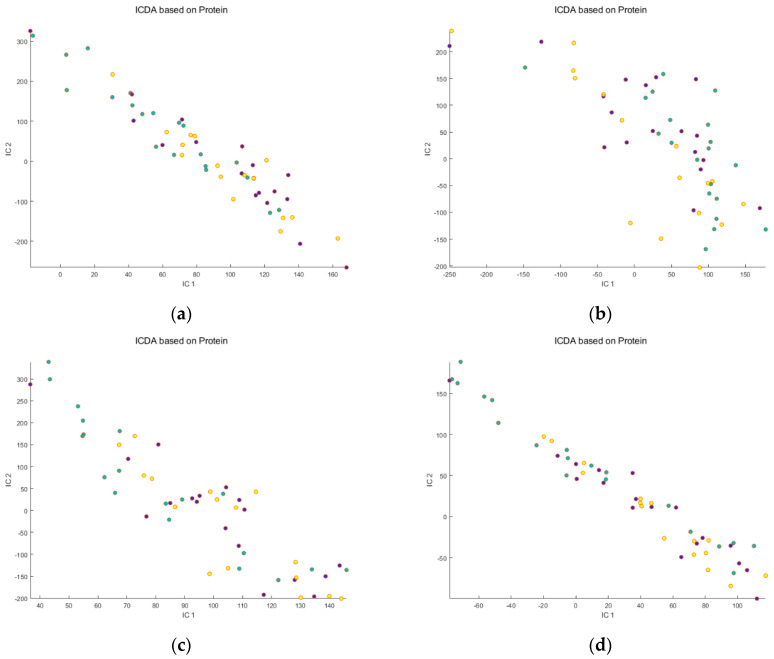
Scores of first 2 ICs for the 4 untreated data sets: (**a**) Raw Reverse Phase; (**b**) Raw HILIC; (**c**) Osmolality−corrected Reverse Phase; (**d**) Osmolality−corrected HILIC. Legend: 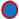 Protein−free group, 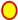 Zein group, 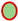 WPI group. Note that no separation between the groups of consumed proteins was observed, for either raw or osmolality-corrected RP and HILIC data sets.

**Figure 2:**
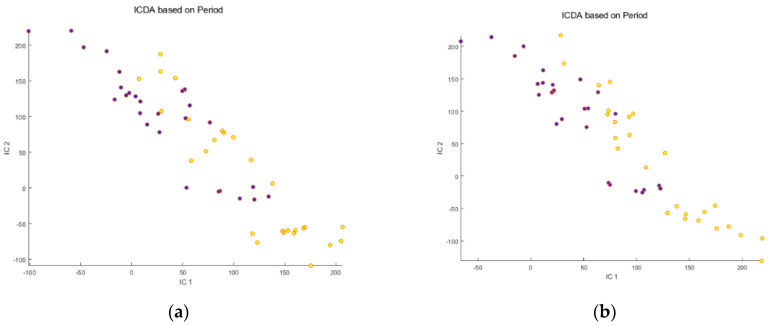

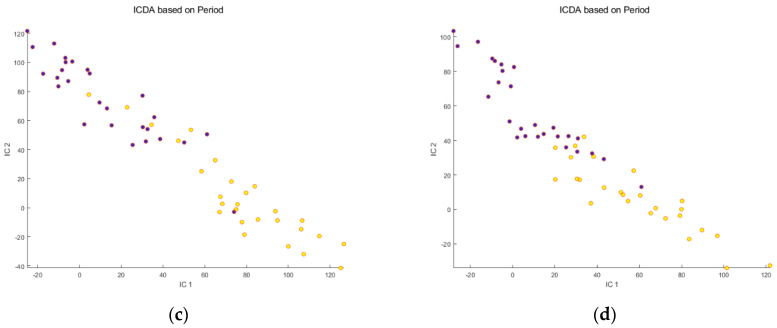
Scores of the first 2 ICs for the 4 untreated data sets: (**a**) Raw Reverse Phase; (**b**) Osmolality−corrected Reverse Phase; (**c**) Raw HILIC; (**d**) Osmolality−corrected HILIC. Legend: 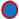 Before meal ingestion, 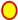 9 h after meal ingestion. Note that osmolality correction did not significantly reduce the dispersion of the individuals, nor increase the separation between the “before” and “after” meal ingestion groups.

We investigated a pre-data acquisition calibration strategy based on osmolality adjustment and a post-acquisition mathematical correction for their feasibilities to overcome sample concentration variability. First, the IC-DA was applied to each data set (raw RP, osmolality-corrected RP, raw HILIC and osmolality-corrected HILIC) without mathematical correction, to evaluate the dispersion of the Scores and to evaluate the quality of the separation of the groups of samples. Two grouping criteria were tested: the nature of the proteins present in the meals (no protein group, WPI group and zein group), and the period (“before meal ingestion” vs. “after meal ingestion”). No separation of the groups was observed for the protein groups (Figure 1a–d). In contrast, a partial separation was observed for “before” vs. “after” meal ingestion (Figure 2a–d). The osmolality correction of the RP and HILIC samples did not significantly reduce the dispersion of the individuals, nor increase the separation of the “before meal ingestion” and “after meal ingestion” periods (Figure 2b,d).

Permutation tests were used to calculate the correct classification rates for the three protein groups for IC-DA applied to progressively more and more permuted groups. Figure S1 shows that the proportion of correct classification for the true groups is not better than for the permuted groups.

**Figure 3:**
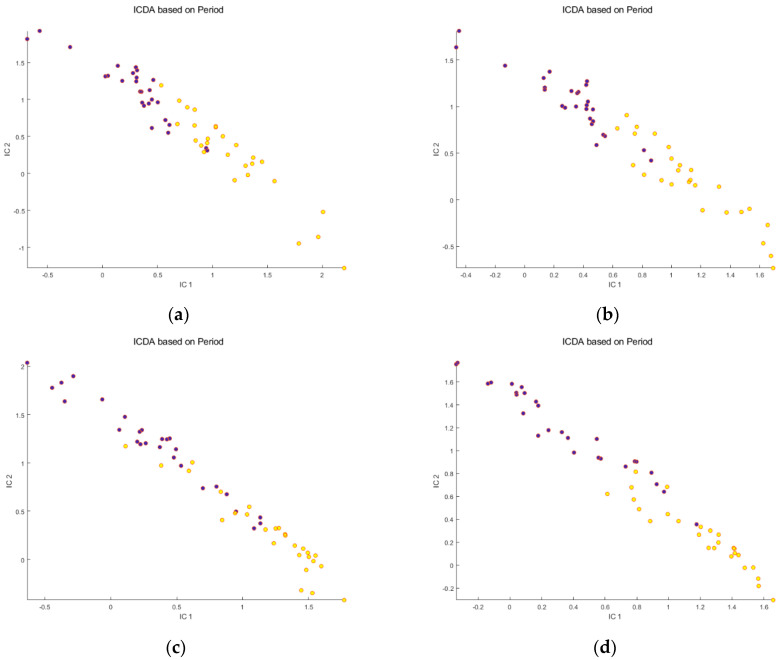
Scores of the first 2 ICs for the 4 untreated data sets after SNV correction of the signals: (**a**) Raw Reverse Phase; (**b**) Osmolality−corrected Reverse Phase; (**c**) Raw HILIC; (**d**) Osmolality−corrected HILIC. Legend: 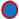 Before meal ingestion, 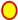 9 h after meal ingestion. Note that SNV correction improves the separation between the groups and decreases the dispersion of the samples within each group.

**Figure 4:**
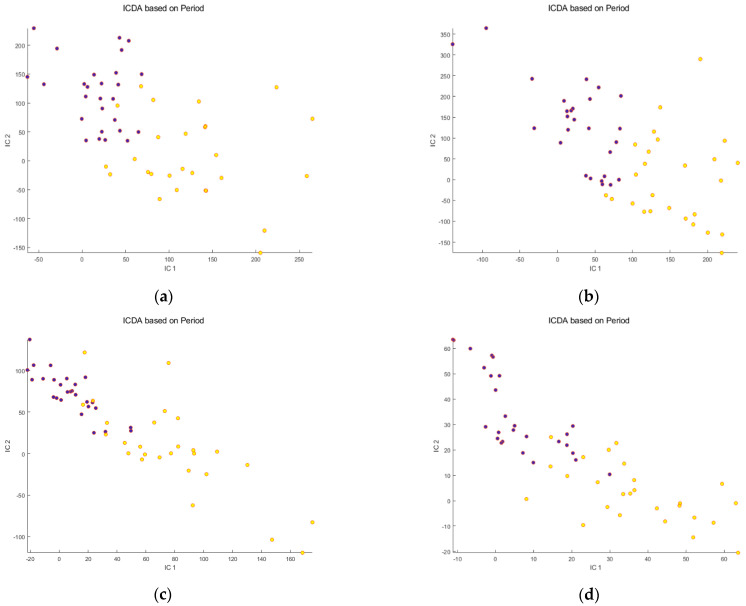
Scores of the first 2 ICs for the 4 data sets after PQN correction of the signals: (**a**) Raw Reverse Phase; (**b**) Osmolality−corrected Reverse Phase; (**c**) Raw HILIC; (**d**) Osmolality−corrected HILIC. Legend: 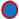 Before meal ingestion, 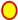 9 h after meal ingestion. Note that PQN correction increases the dispersion of the samples within the groups.

Secondly, the IC-DA was applied to each data set after mathematical correction of the signals. For all data sets, SNV (Figure 3a–d) increased the separation between the “before meal ingestion” and “after meal ingestion” periods, while decreasing the dispersion of the samples within each group. The improvement was greater for the osmolality-corrected data sets. On the other hand, PQN (Figure 4a–d), while slightly improving the separation of the groups, increased the dispersion of the samples. 

### 3.2. AComDim-ICA

In order to determine whether the osmolality correction and SNV pretreatment has a significant effect on the separation of the samples due to the different factors, AComDim-ICA was applied to both the two SNV-treated, osmolality-corrected data sets and to the non-SNV-pretreated data without osmolality correction.

The initial RP data without osmolality adjustment contained 1350 features, and 840 features after elimination of variables not present in the QCs and intense in the blanks. The RP matrix with osmolality adjustment contained 1117 features, and 562 after elimination of variables not present in the QCs and intense in the blanks. The HILIC matrix without osmolality adjustment contained 8932 features, and 2563 after elimination of variables not present in the QCs and intense in the blanks. The initial HILIC data with osmolality adjustment consisted of 10,160 features. After elimination of variables not present in the QCs and intense in the blanks, the matrix contained 2943 variables. The initial matrices are reported in Table S1.

**Figure 5:**
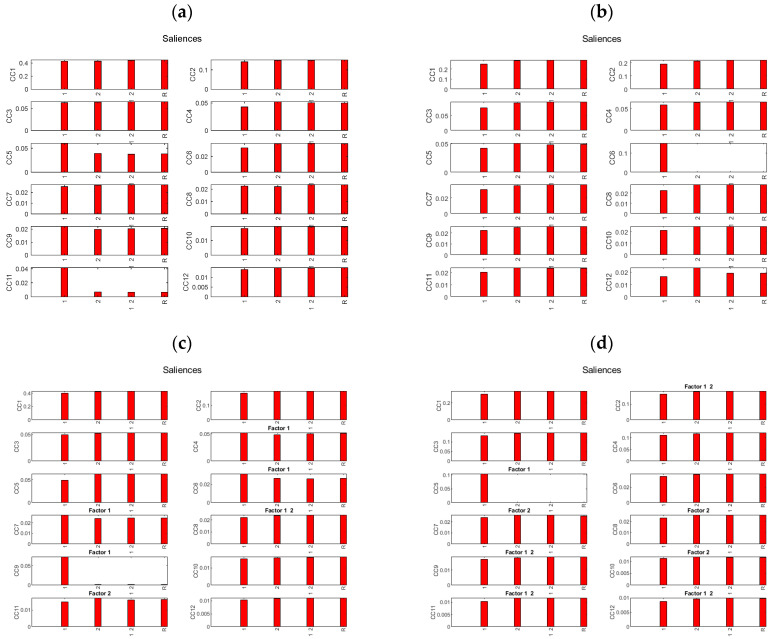
Saliences for the AComDim-ICA tables corresponding to Factor1 (Period: before and after meal), Factor2 (Protein type: no protein group, WPI−group and zein group), Factor12 (interaction between Factor1 and Factor2) and the residuals. AComDim-ICA was applied to SNV−pretreated, osmolality−corrected (**a**) RP data, (**b**) HILIC data; and to the raw (**c**) RP data, and (**d**) HILIC data. Note that only Factor1 contributes to one of the CCs (CC 11 and CC 9 for RP data, and CC 6 and CC 5 for HILIC data), while Factor2 and Factor12 do not contribute to any of the CCs.

The plots of the saliences show the contribution of each table, corresponding to Factor1 (Period: before and after meal), Factor2 (Protein type: no protein group, WPI-group and zein-group), Factor12 (the interaction between Factor1 and Factor2) and the residuals for the 12 Common Components (CC) (Figure 5a–d).

For both RP and HILIC data, only Factor1 contributes to one of the CCs (CC 11 and CC 9 for RP data, and CC 6 and CC 5 for HILIC data, Figure 4) while Factor2 and Factor12 do not contribute to any of the CCs, thus indicating that the other CCs only contain noise from the residuals. As well, for the HILIC data, Factor1 contributes to earlier CCs than for the RP data, indicating that the HILIC data set contains more variability from Factor1 than does the RP data set. One can also see that in both cases, the salience of the Factor1 table on CC 1 is only slightly lower than that of the residuals table, indicating that the Factor1 tables do not contain very much variability different from that due to the noise in the residuals.

**Figure 6:**
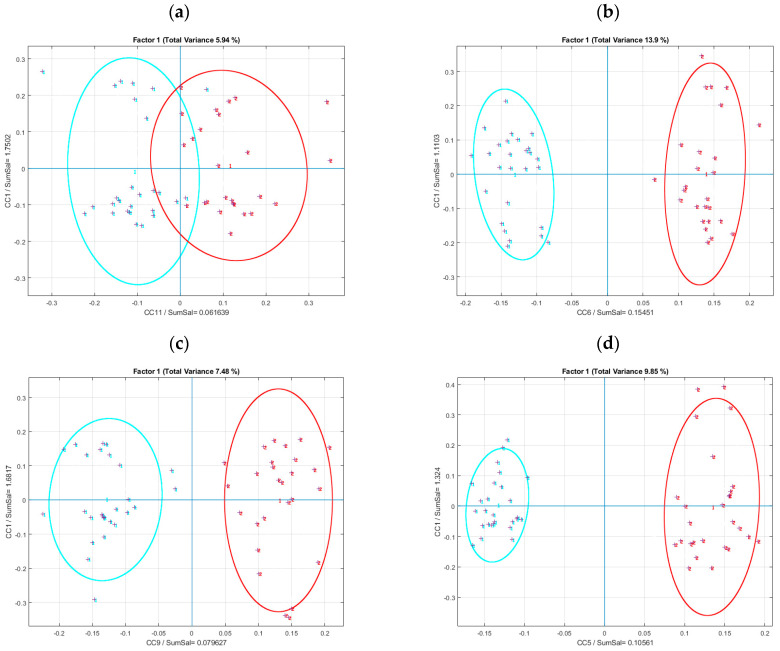
Global Scores given by AComDim-ICA (**a**) CC 11 plotted against CC 1 for the osmolality-corrected, SNV-pretreated RP data; (**b**) CC 6 plotted against CC 1 for the osmolality-corrected, SNV-pretreated HILIC data; (**c**) CC 9 plotted against CC 1 for the non-corrected, raw RP data; (**d**) CC 5 plotted against CC 1 for the non-corrected raw HILIC data. The colors for the Scores for Factor1 are 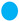: Before meal ingestion ; 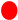: After meal ingestion. Note the better separation of the groups for non-corrected, raw RP data, whereas for HILIC there is little difference between osmolality-corrected, SNV-pretreated data and the non-corrected, raw data.

The application of AComDim-ICA separated the “before” and “after” the meal ingestion groups along CC 11 and CC 9 (Figure 6a,c) for the RP data, and along the CC 6 and CC 5 for the HILIC data (Figure 6b,d). The data obtained by HILIC give clearer separations between the groups than do the RP data. The Scores of CC 11 and CC 9 (for RP data) and CC 6 and CC 5 (for HILIC data) plotted against CC 1, which is due to the noise in the residuals table, confirms the better separation with the HILIC data (Figure 6a–d). It is interesting to note that for the RP data, the separation of the two groups is better for the raw, non-corrected data than for the SNV-pretreated, osmolality-corrected data (Figure 6a,c), while there is little difference for the HILIC data (Figure 6a,c).

The CC 1 saliences were used to calculate an F-value as an estimate of the significance of the factors (Figure S2a–d). In Figure 6, the Factor1 F-value for the SNV-pretreated, osmolality-corrected RP data is very slightly greater than 1.04, while the value for the raw, non-corrected data is about 1.1. The Factor1 F-value for the SNV-pretreated, osmolality-corrected HILIC data is 1.17, while the value for the raw, non-corrected data is below 1.13. These values are far from the 95% confidence level, so to confirm the significance of Factor1, an AComDim-ICA permutation test was performed on the four data sets and the results are presented in Figure S3.

The permutation tests were used to calculate the salience-based F values for AComDim_ICA applied to progressively more and more permuted factor levels. If a factor is really significant, its F-value for the true factor levels should be greater than that for the permuted levels, and the F-value should decrease as the extent of the permutations increases. This is what is observed in Figure S3 for Factor1 (Period: before and after meal), but neither for Factor2 (Protein type: no protein, WPI and zein), nor for Factor12 (the interaction between Factor1 and Factor2).

The most important variables for the separation of the levels of Factor1 were determined using an S-plot based on the correlations and covariances calculated between the SNV-treated, osmolality-corrected RP data and the Scores for CC 11, and between the SNV-treated, osmolality-corrected HILIC data and the Scores for CC 6; as well as the raw, non-corrected RP data and the Scores for CC 9, and between the raw, non-corrected HILIC data and the Scores for CC 5. The S-plots are presented in Figure S4 and the selected variables are plotted in Figure S5. A *t*-test was then applied to compare the intensities of the variables selected by the S-plot in the two data sets and confirmed their significance. 

### 3.3. Discriminant Metabolites

The variables selected by S-plot were submitted to a *t*-test in order to determine the ones significantly different between the “before” and “after” meal ingestion groups. For the RP data without osmolality adjustment, nine features were selected by S-plot, of which seven were shown to be significantly different (*p* < 0.05) by a *t*-test. For the RP data with osmolality and SNV pretreatment, the S-plot selected 13 features, of which 11 were shown to be significantly different (*p* < 0.05). For the HILIC data without osmolality adjustment, 20 features were selected by the S-plot, of which 13 were shown to be significantly different (*p*-value < 0.05). For the HILIC data with osmolality adjustment and SNV pretreatment, the S-plot selected 24 features, of which 21 were shown to be significantly different (*p* < 0.05).

**Figure 7:**
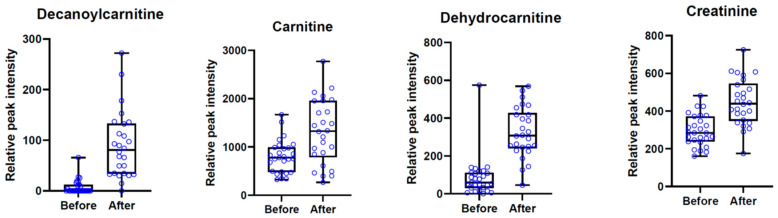

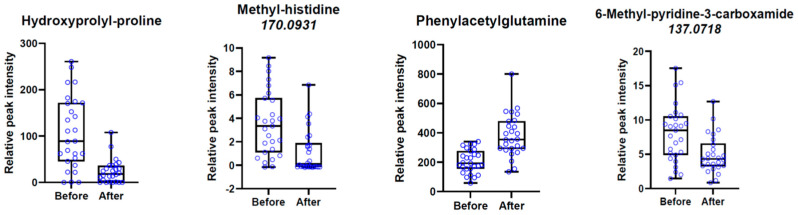
Boxplot of signal intensities of identified metabolites before and 9 h after meal intake.

Thus, AComDim-ICA applied to HILIC data with osmolality adjustment and SNV treatment permitted identifying the greatest number of features significantly different between the “before” and “after” meal ingestion groups. We performed MS-MS experiments to identify these 21 compounds. Detailed information on metabolite identification is reported in Table S2. Amongst them, eight were identified with the confidence level 1 or 2, according to the Metabolomics Standards Initiative [20]. The intensities of identified metabolites are presented in Figure 7. The intensities of non-identified metabolites are presented in Figure S6. Nine hours after meal intake, higher urinary excretion of creatinine, carnitine, dehydrocarnitine, decanoylcarnitine and phenylacetylglultamine (*p* < 0.001) was observed, whereas the excretion of hydroxyprolyl-proline, 3-methylhistidine and 6-methyl-pyrimidine-3-carboxamide decreased (*p* < 0.001). 

## 4. Discussion

The detection of the metabolites that could discriminate the consumption of highly digestible protein from a low-digestible protein was the main objective of our study. Dietary proteins are hydrolyzed by proteases and peptidases to AAs, dipeptides and tripeptides in the small intestine. Unabsorbed dietary peptides and AAs are mostly excreted in feces while the absorbed AAs are metabolized and their metabolites can be excreted in urine. Thus, we hypothesized that a low-digestible protein would lead to a different urinary metabolic profile than a highly digestible protein. 

Surprisingly, we did not observe any changes in urinary metabolome specifically associated with low or high protein digestibility, even in the absence of any protein in the meal (protein-free diet group). It is possible that an acute meal protocol was not sufficient to observe changes in the urinary metabolome related to the kind of protein intake. Indeed, even if some studies permitted us to detect the metabolites specifically associated with the dietary component after the exposure to a single meal [21], in most studies 3–5 consecutive days of exposure to intervention meals may be needed [22,23]. Unfortunately, we could not reproduce these experimental conditions over a longer period, as it was necessary to prevent the participants from consuming any other source of protein, in order to avoid interactions with the intervention meal. In such a case, the equilibrium in the alimentation of the participants would not have been maintained, as the protein-free and zein-containing meal do not supply indispensable AAs at a level sufficient to cover nutritional needs.

In most metabolomics studies evaluating the effect of protein intake, the focus is given either to vegan vs. omnivorous and/or type of meat consumed in the diet (red meat, poultry, processed meat or fish; [2,23,24]). The metabolites related to veganism have relevance to lipid, carbohydrate metabolism and polyphenols and are considered as biomarkers of wholegrain, vegetables and nuts, but not proteins [1,4,5,7,8,25]. Only the study of Hernadez and al., which focused on the source of protein, identified several plasma metabolites exclusively related to plant protein consumption [8]. These metabolites include AAs, AA derivatives (N-oleoylglycine), some nitrogen-containing compounds (acetylcholine, niacinamide) and different lipid species. In our study, no difference in dispensable AA plasma profile was reported after WPI intake compared to zein and protein-free intake, but the IAA were increased in the plasma of WPI group [11]. However, the protein sources tested in our study were protein isolates, while the changes in metabolome related to protein consumption are generally observed in the context of a complete food protein matrix [1,2,4,6,7,23,24,25]. 

In contrast, we observed changes between the baseline and the 9 h postprandial period. As these changes could not be ascribed to the consumption of proteins, they must be due to the consumption of the meal in which the non-protein part was similar for the groups.

We observed that after meal intake (containing no protein, WPI or zein) the excretion of carnitine, dehydrocarnitine, creatinine, decanoylcarnitine and phenylacetylglutamine increased. They are known to be associated with animal products: carnitine and creatinine are related to meat and dairy products. The urinary excretion of medium-chain acylcarnitines may be influenced by red meat [23]. In addition, urinary phenylacetylglutamine, a microbial-derived proteolysis product, is specifically associated with red meat intake [10,25]. All these compounds are human metabolites and besides their supply from the diet, they may be synthesized from endogenous AAs. In contrast, the urinary concentrations of hydroxyprolyl-proline, 3-methylhisitidine and 6-methyl-pyrimidine-3-carboxamide (*N*-methylnicotinamide) were decreased in all groups. *N*-methylnicotinamide is the metabolite of niacin (vitamin B), and its excretion is influenced by the dietary intake of foods that are the sources of this vitamin, such as eggs, fish, meat, dairy products and poultry. Hydroxyprolyl-proline is a collagen-derived dipeptide, and urinary 3-methylhistidine is associated with the intake of red meat and poultry [10,24]. Thus, the lower excretion of these metabolites is in accordance with the fact that the test meals did not contain collagen or other meat proteins. Even if one of the test meals used for this study contained AP, it was the whey protein isolate, and it could not provide the sufficient supply of niacin. 

Another question of our study was to investigate a pre-data acquisition calibration strategy based on osmolality adjustment and a post-acquisition mathematical correction for their feasibility to overcome sample concentration variability. Urine samples present large variations in the concentrations of endogenous metabolites, which are caused by diverse factors such as water consumption, food consumption and lifestyle. In our study, additional variability could be introduced by experimental conditions, as our samples were not 24 h urines. The pre-acquisition osmolality-based dilution was reported to be efficient to eliminate such deviations, and the combination of osmolality adjustment and the subsequent mathematical normalization enhances the data quality even further [26,27,28,29]. Thus, we applied the subsequent chemometrical analysis to the two SNV-treated, osmolality-corrected data sets after removal of the blanks and QCs.

Before data acquisition, one batch was prepared where the osmolality was equivalent for the samples, and the second batch contained the samples without adjustment to the lowest osmolality value. The Independent Components Discriminant Analysis showed the beneficial effect of osmolality adjustment combined with SNV pretreatment on reducing the dispersion and improving the separation of the samples for the levels of Factor1. However, the separation between the “before” and “after” meal consumption groups was also observed for the raw data, without osmolality or SNV correction. AComDim_ICA, including a permutation test, confirmed that only Factor1 had an effect on the metabolites of the individuals. It also allowed the detection of those metabolites affected. AComDim_ICA also showed that in the case of the HILIC data, the SNV pretreatment and osmolality correction improved the separation between the groups, but not in the case of the RP data. Moreover, the matrix used for AComDim_ICA contained more features after SNV pretreatment and osmolality correction for both HILIC and RP data, as compared to the data sets without osmolality adjustment and without SNV pretreatment. The number of features detected by S-plot and the number of features significantly differing between the groups were also greater after SNV pretreatment and osmolality correction. These results are in agreement with previous studies that showed the importance of normalization strategy for urinary metabolome profiling [26,27,28,29]. Thus, we may conclude that the detection of variables related to meal consumption is possible in urine samples without osmolality-based dilution, but the normalization may contribute to a better data quality.

The strength of this study is that it was conducted under highly controlled interventional conditions: the intervention meal was the only dietary source for the participants during that day. In principle, the experimental design used should have permitted us to evaluate the specific effects of low- and highly digestible proteins. In practice, the single intake of a meal was not sufficient to produce a significant modification of the metabolism. This is the main limitation of the study, as the changes in metabolites could have been more pronounced in the case of longer-term exposure. Another limitation is that the habitual dietary style of the subjects was not taken into account. Even if the volunteers followed dietary advice during the week before the experiment to homogenize the level of protein intake, various foods could be chosen among a list. Thus, interactions with prior diet are not to be excluded. 

## 5. Conclusions

After one acute intake of low/highly digestible protein corresponding to plant or animal origin, respectively, no specific metabolites could be detected in urine, and longer-term exposure may be needed. Some changes in metabolites were observed 9 h after meal intake independently of the group, with an increase in carnitine, dehydrocarnitine, creatinine and acylcarnitines. This suggests that the excretion products do not come from dietary proteins, but from endogenous synthesis related to meal digestion and metabolization. The normalization strategy of osmolality adjustment combined with SNV pretreatment showed the beneficial effect on reducing the dispersion and improving the separation of the samples and the detection of a more important number of discriminate metabolites. AComDim_ICA applied to the RP and HILIC data was able to separate the “before” and “after” meal consumption groups and identify variables that varied significantly, whether the data were osmolality-corrected and SNV-pretreated or not.

## Data Availability

The data presented in this study are available on request from the corresponding author due to ethical reasons.

## Supplementary Materials

The following supporting information can be downloaded at: https://www.mdpi.com/article/10.3390/metabo14040177/s1, Figure S1: Proportion of correct classification for IC-DA permutation test on untreated data for protein groups; Figure S2: F-values calculated as the ratio of the salience of the residual table for CC 1 on the saliences of the other tables; Figure S3: Proportion of correct classification for AComDim-ICA permutation test on SNV-treated data for period groups for the original and the permuted groups, for the three factors; Figure S4: S-plot for SNV-treated data for “before meal ingestion” and “after meal ingestion” groups; Figure S5: Variables selected by S-plot for SNV-treated data for “before meal ingestion” and “after meal ingestion” groups. Figure S6: Boxplot of signal intensities of non-identified metabolites before and 9 h after meal intake; Table S1: The initial matrices of RP and HILIC data. Table S2: MS-MS characteristics of urinary metabolites related to the consumption of experimental meal.

## References

[1] Schmidt J.A., Rinaldi S., Ferrari P., Carayol M., Achaintre D., Scalbert A., Cross A.J., Gunter M.J., Fensom G.K., Appleby P.N. (2015). Metabolic profiles of male meat eaters, fish eaters, vegetarians, and vegans from the EPIC-Oxford cohort. Am. J. Clin. Nutr..

[2] Schmidt J.A., Fensom G.K., Rinaldi S., Scalbert A., Gunter M.J., Holmes M.V., Key T.J., Travis R.C. (2021). NMR Metabolite Profiles in Male Meat-Eaters, Fish-Eaters, Vegetarians and Vegans, and Comparison with MS Metabolite Profiles. Metabolites.

[3] Hovinen T., Korkalo L., Freese R., Skaffari E., Isohanni P., Niemi M., Nevalainen J., Gylling H., Zamboni N., Erkkola M. (2021). Vegan diet in young children remodels metabolism and challenges the statuses of essential nutrients. EMBO Mol. Med..

[4] Miles F.L., Orlich M.J., Mashchak A., Chandler P.D., Lampe J.W., Duerksen-Hughes P., Fraser G.E. (2022). The Biology of Veganism: Plasma Metabolomics Analysis Reveals Distinct Profiles of Vegans and Non-Vegetarians in the Adventist Health Study-2 Cohort. Nutrients.

[5] Miles F.L., Lloren J.I.C., Haddad E., Jaceldo-Siegl K., Knutsen S., Sabate J., Fraser G.E. (2019). Plasma, Urine, and Adipose Tissue Biomarkers of Dietary Intake Differ Between Vegetarian and Non-Vegetarian Diet Groups in the Adventist Health Study-2. J. Nutr..

[6] Xu J., Yang S., Cai S., Dong J., Li X., Chen Z. (2010). Identification of biochemical changes in lactovegetarian urine using ^1^H NMR spectroscopy and pattern recognition. Anal. Bioanal. Chem..

[7] Lindqvist H.M., Rådjursöga M., Torstensson T., Jansson L., Ellegård L., Winkvist A. (2021). Urine Metabolite Profiles and Nutrient Intake Based on 4-Day Weighed Food Diary in Habitual Vegans, Vegetarians, and Omnivores. J. Nutr..

[8] Hernández-Alonso P., Becerra-Tomás N., Papandreou C., Bulló M., Guasch-Ferré M., Toledo E., Ruiz-Canela M., Clish C.B., Corella D., Dennis C. (2020). Plasma Metabolomics Profiles are Associated with the Amount and Source of Protein Intake: A Metabolomics Approach within the PREDIMED Study. Mol. Nutr. Food Res..

[9] Gaudichon C., Calvez J. (2021). Determinants of amino acid bioavailability from ingested protein in relation to gut health. Curr. Opin. Clin. Nutr. Metab. Care.

[10] Khodorova N.V., Jouan-Rimbaud D., Pilard S., Cordella C., Locquet N., Oberli M., Gaudichon C. (2022). Consumption of Boiled, but Not Grilled, Roasted, or Barbecued Beef Modifies the Urinary Metabolite Profiles in Rats. Mol. Nutr. Food Res..

[11] Calvez J., Benoit S., Piedcoq J., Khodorova N., Azzout-Marniche D., Tomé D., Benamouzig R., Airinei G., Gaudichon C. (2021). Very low ileal nitrogen and amino acid digestibility of zein compared to whey protein isolate in healthy volunteers. Am. J. Clin. Nutr..

[12] Barnes R.J., Dhanoa M.S., Lister S.J. (1989). Standard normal variate transformation and de-trending of near-infrared diffuse reflectance spectra. Appl. Spectrosc..

[13] Barnes R.J., Dhanoa M.S., Lister S.J. (1993). Correction to the Description of Standard Normal Variate (SNV) and De-Trend (DT) Transformations in Practical Spectroscopy with Applications in Food and Beverage Analysis—2nd Edition. J. Near Infrared Spectrosc..

[14] Dieterle F., Ross A., Schlotterbeck G., Senn H. (1993). Probabilistic quotient normalization as robust method to account for dilution of complex biological mixtures. Application in ^1^H NMR metabonomics. Anal. Chem..

[15] Habchi B., Alves S., Jouan-Rimbaud Bouveresse D., Moslah B., Paris A., Lécluse Y., Gauduchon P., Lebailly P., Rutledge D.N., Rathahao-Paris E. (2017). An innovative chemometric method for processing direct introduction high resolution mass spectrometry metabolomic data: Independent component–discriminant analysis (IC–DA). Metabolomics.

[16] Harrington P.B., Vieira N.E., Espinoza J., Nien J.K., Romero R., Yergey A.L. (2005). Analysis of variance-principal component analysis: A soft tool for proteomic discovery. Anal. Chim. Acta.

[17] Smilde A.K., Jansen J.J., Hoefsloot H.C., Lamers R.J., van der Greef J., Timmerman M.E. (2005). ANOVA-simultaneous component analysis (ASCA): A new tool for analyzing designed metabolomics data. Bioinformatics.

[18] Qannari M., Wakeling I., Courcoux P., MacFie H.J.H. (2000). Defining the underlying sensory dimensions. Food Qual. Prefer..

[19] Tormena C.D., Rutledge D.N., Rakocevic M., Bruns R.E., Scarminio I.S., Marcheafave G.G., Pauli E.D. (2022). Exogenous application of bioregulators in Coffea arabica beans during ripening: Investigation of UV–Visible and NIR mixture design-fingerprints using AComDim-ICA. Microchem. J..

[20] Sumner L.W., Amberg A., Barrett D., Beale M.H., Beger R., Daykin C.A., Fan T.W., Fiehn O., Goodacre R., Griffin J.L. (2007). Proposed minimum reporting standards for chemical analysis Chemical Analysis Working Group (CAWG) Metabolomics Standards Initiative (MSI). Metabolomics.

[21] Heinzmann S.S., Brown I.J., Chan Q., Bictash M., Dumas M.E., Kochhar S., Stamler J., Holmes E., Elliott P., Nicholson J.K. (2010). Metabolic profiling strategy for discovery of nutritional biomarkers: Proline betaine as a marker of citrus consumption. Am. J. Clin. Nutr..

[22] Wilson T., Garcia-Perez I., Posma J.M., Lloyd A.J., Chambers E.S., Tailliart K., Zubair H., Beckmann M., Mathers J.C., Holmes E. (2019). Spot and Cumulative Urine Samples Are Suitable Replacements for 24-Hour Urine Collections for Objective Measures of Dietary Exposure in Adults Using Metabolite Biomarkers. J. Nutr..

[23] Wedekind R., Keski-Rahkonen P., Robinot N., Mercier F., Engel E., Huybrechts I., Scalbert A. (2020). Signatures of 10 Processed and Non-processed Meat Products after In Vitro Digestion. Metabolites.

[24] Cheung W., Keski-Rahkonen P., Assi N., Ferrari P., Freisling H., Rinaldi S., Slimani N., Zamora-Ros R., Rundle M., Frost G. (2017). A metabolomic study of biomarkers of meat and fish intake. Am. J. Clin. Nutr..

[25] Meslier V., Laiola M., Roager H.M., De Filippis F., Roume H., Quinquis B., Giacco R., Mennella I., Ferracane R., Pons N. (2020). Mediterranean diet intervention in overweight and obese subjects lowers plasma cholesterol and causes changes in the gut microbiome and metabolome independently of energy intake. Gut.

[26] Vogl F.C., Mehrl S., Heizinger L., Schlecht I., Zacharias H.U., Ellmann L., Nürnberger N., Gronwald W., Leitzmann M.F., Rossert J. (2016). Evaluation of dilution and normalization strategies to correct for urinary output in HPLC-HRTOFMS metabolomics. Anal. Bioanal. Chem..

[27] Chetwynd A.J., Abdul-Sada A., Holt S.B., Hill E.M. (2016). Use of a pre-analysis osmolality normalisation method to correct forvariable urine concentrations and for improved metabolomic analyses. J. Chrom. A.

[28] Gagnebin Y., Tonoli D., Lescuyer P., Ponte B., De Seigneux S., Martin P.Y., Schappler J., Boccard J., Rudaz S. (2017). Metabolomic analysis of urine samples by UHPLC-QTOF-MS: Impact of normalization strategies. Anal. Chim. Acta.

[29] Hertel J., Rotter M., Frenzel S., Zacharias H.U., Krumsiek J., Rathkolb B., Hrabe de Angelis M., Rabstein S., Pallapies D., Brüning T. (2018). Dilution correction for dynamically influenced urinary analyte data. Anal. Chim. Acta.

