# Supplementary material for: Urine Metabolite Profiles after the Consumption of a Low- and a High-Digestible Protein Meal, and Comparison of Urine Normalization Techniques
Source: Metabolites. 2024 Mar 22;14(4):177. doi: 10.3390/metabo14040177 (PMC11052258; doi:10.3390/metabo14040177)
Supplement: Supplementary file 1 [file metabolites-14-00177-s001.zip › Proteos_Supplementary_Data_Metabolites_soumission2_revised_08032024.pdf]

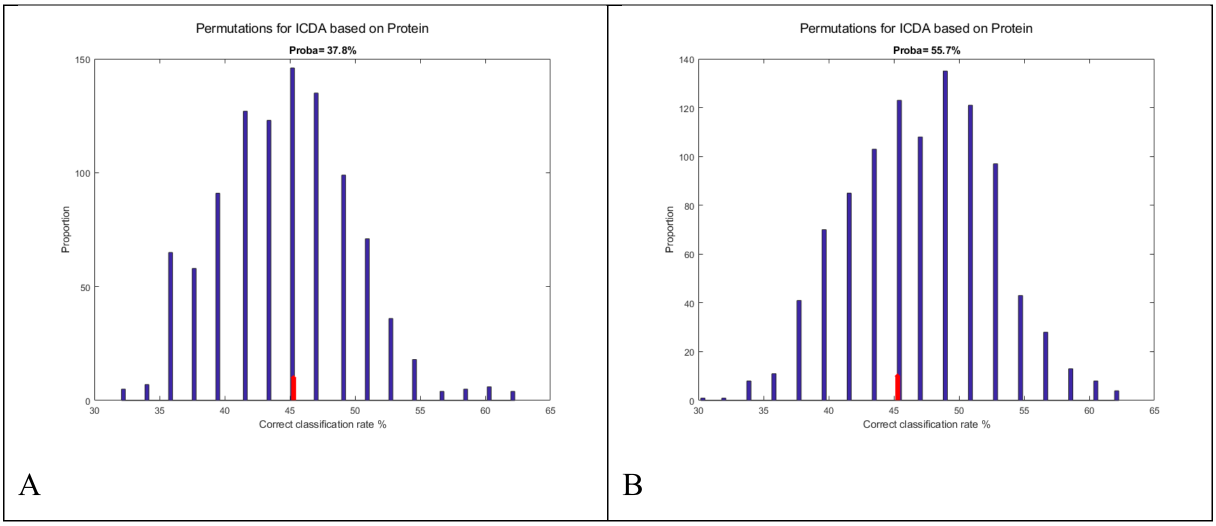

Figure S1: Proportion of correct classification for IC-DA permutation test on untreated data for Protein groups : A) Osmolality-corrected Reverse Phase; B) Osmolality-corrected HILIC; Legend: Blue bars, permuted groups; Red bar, true groups.

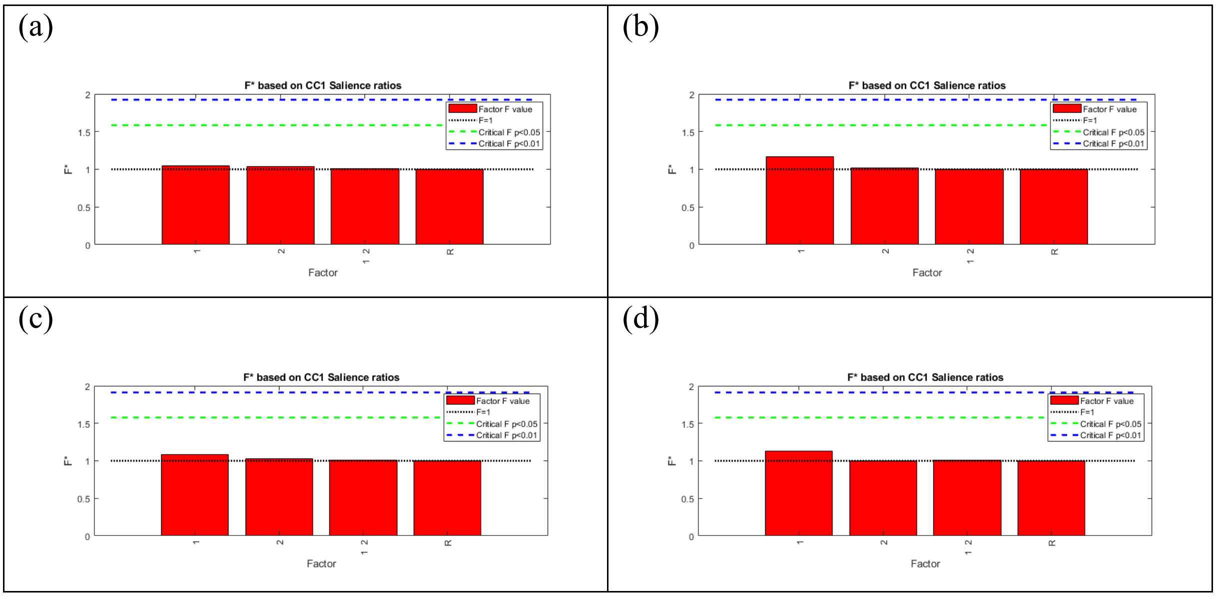

Figure S2. F-values calculated as the ratio of the salience of the residual table for CC 1 on the saliences of the other tables. (a) Osmolality-corrected, SNV-pretreated RP data; (b) Osmolality-corrected, SNV-pretreated HILIC data, (c) non-corrected, raw RP data; (d) non-corrected, raw HILIC data. Note that the F* value is slightly higher for the non-corrected, raw RP data.

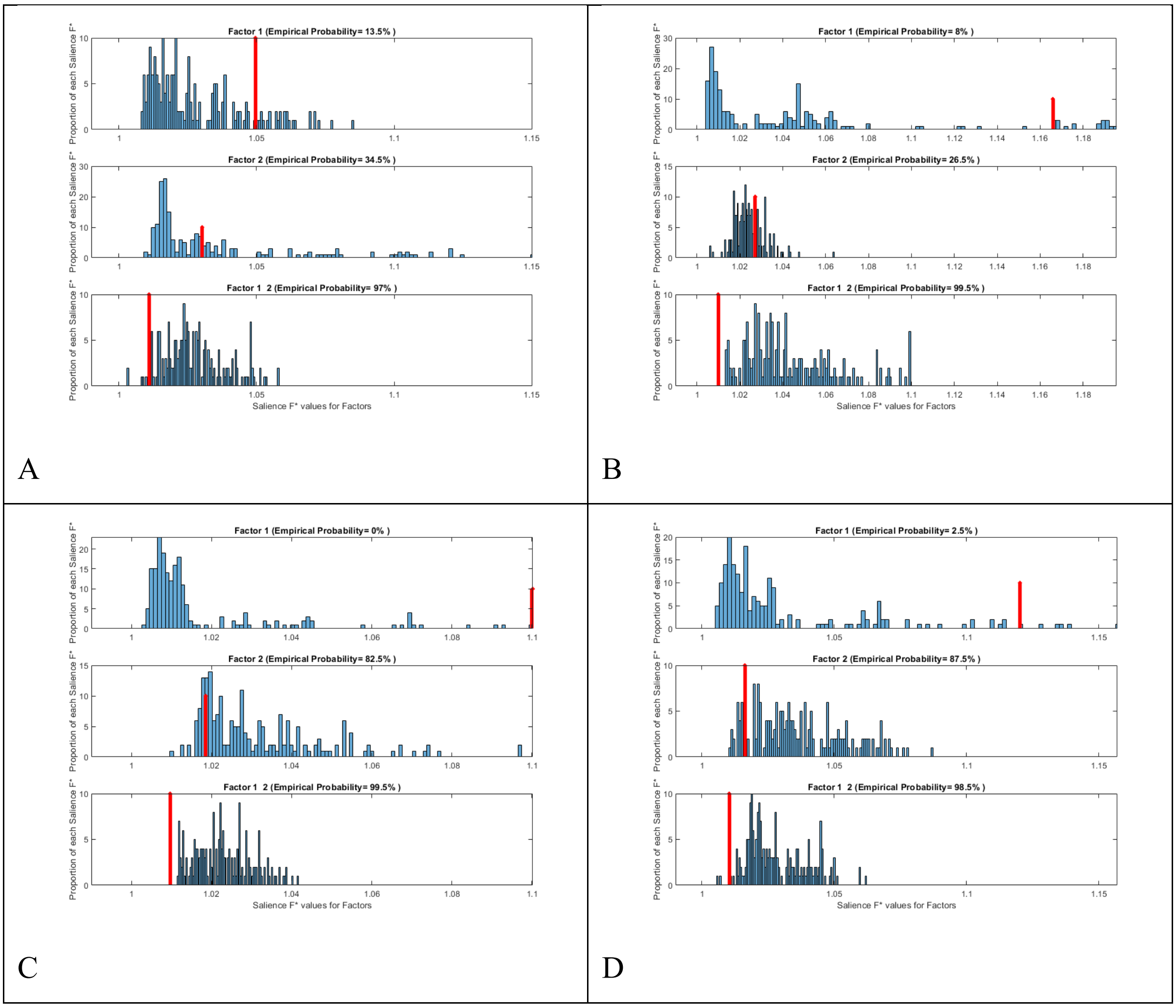

Figure S3: Proportion of correct classification for AComDim-ICA permutation test on the data for Period groups for the original (Red) and the permuted (Blue) groups, for the 3 Factors : A) SNV-treated osmolality-corrected Reverse Phase; B) SNV-treated osmolality-corrected HILIC, C) raw, non-corrected Reverse Phase; D) raw, non-corrected HILIC.

In Figure S3 it is clear that the Factor1 effect is more significant for the HILIC data than for the RP data. In no case are Factor2 and Factor12 significant.

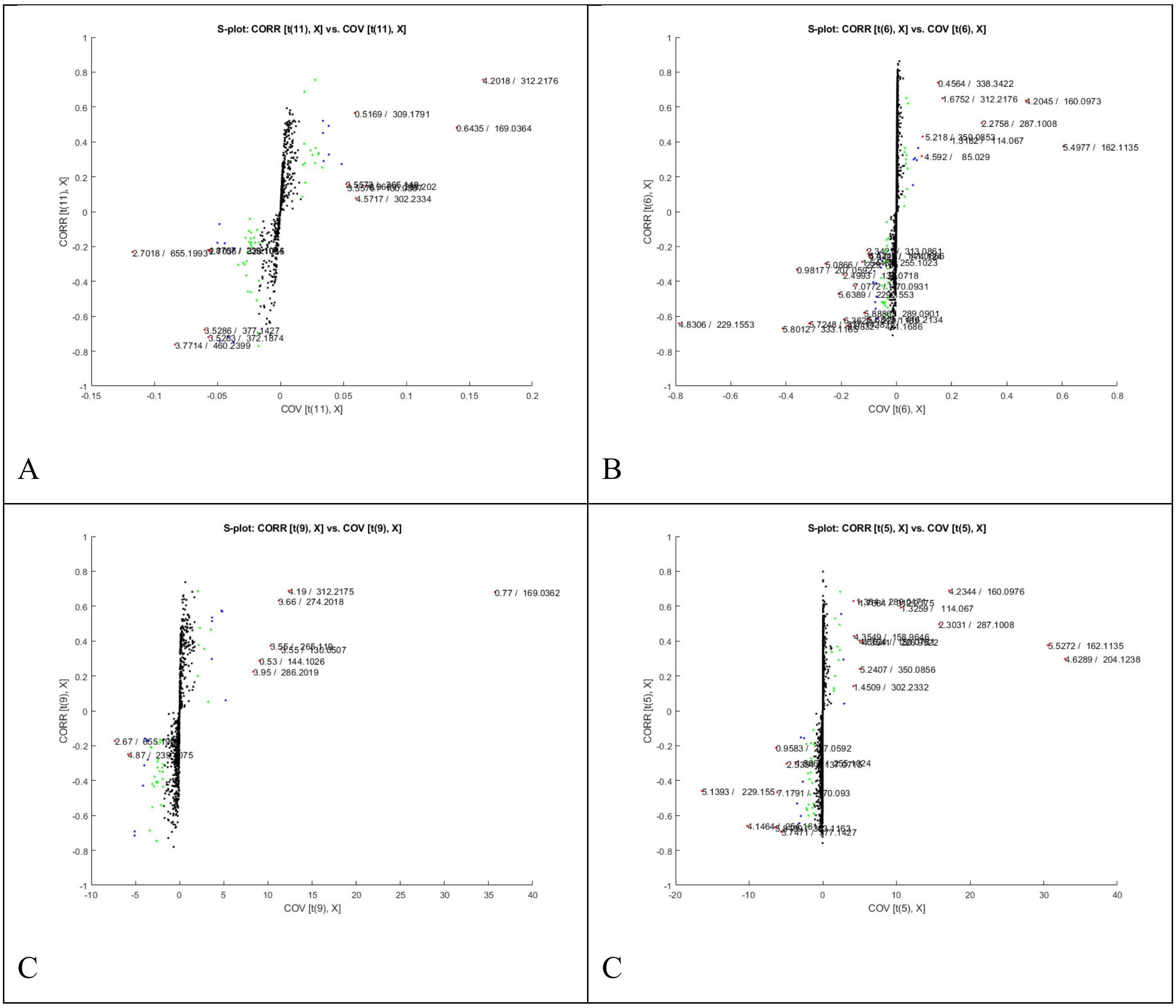

Figure S4: S-plot for data for “before meal ingestion” and “after meal ingestion” groups : A) Based on AComDim-ICA Scores of CC11 for osmolality-corrected, SNV-treated Reverse Phase; B) Based on AComDim-ICA Scores of CC6 for osmolality-corrected, SNV-treated HILIC; C) Based on AComDim-ICA Scores of CC9 for non-corrected, raw Reverse Phase; B) Based on AComDim-ICA Scores of CC5 for non-corrected, raw HILIC.

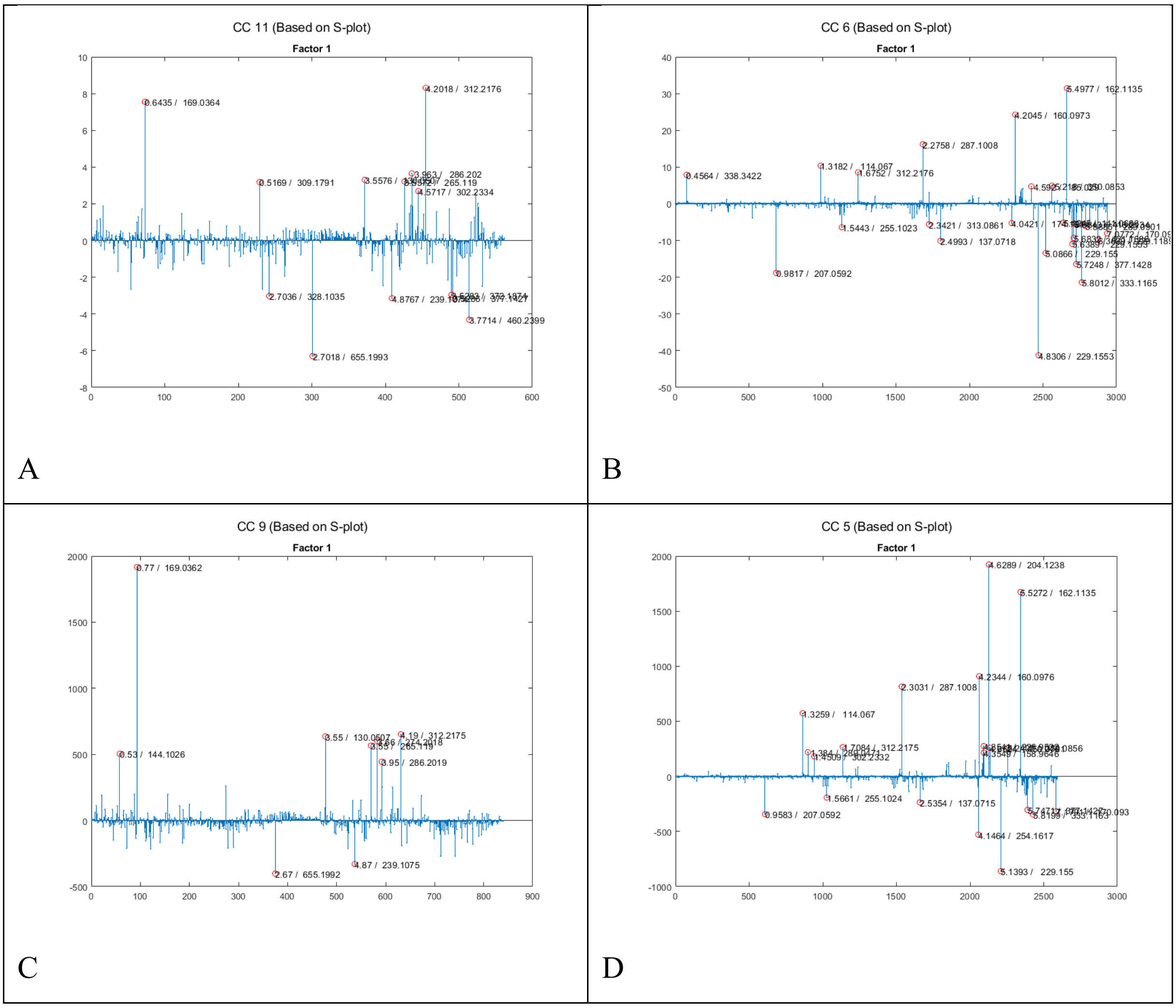

Figure S5: Variables selected by S-plot for “before meal ingestion” and “after meal ingestion” groups : A) Based on AComDim-ICA Scores of CC11 for osmolality-corrected, SNV-treated Reverse Phase; B) Based on AComDim-ICA Scores of CC6 for osmolality-corrected, SNV-treated HILIC; C) Based on AComDim-ICA Scores of CC9 for non-corrected, raw Reverse Phase; B) Based on AComDim-ICA Scores of CC5 for non-corrected, raw HILIC.

Table S2. MS-MS characteristics of urinary metabolites related to the consumption of experimental meal. For some features no MS-MS fragmentation could be obtained due to the low intensity of the ion in the spectrum.

Experim. m/z	Suggested ion	Elemental composition	MS/MS fragment ions	Collision energy, eV	annotation
338.3422	--	C12H44NO	No MS-MS fragments obtained	--	unknown
114.067	$[M+H]^+$	C4H8N3O	86.0848	30	creatinine
312.2176	$[M+H]^+$	C17H30NO4	253.1445, 151.1120, 123.1162, 85.0289, 81.0703	20	decanoylcarnitine
287.1008	$[M+Na]^+$	$C_{13}H_{17}N_2O_4$	136.0765, 130.0506, 91.0537, 84.0446	15	phenylacetylglutamine
229.1553	$[M+H]^+$	C11H21N2O3	142.0866, 70.0654	20	unknown
162.1135	$[M+H]^+$	C7H16NO3	102.0919, 103.0324, 85.0233	15	Carnitine
229.1189	$[M+H]^+$	C10 H17 N2 O4	132.0658, 86.0606	20	Hydroxyprolyl-proline
350.0853	--	--	No MS-MS fragments obtained	--	unknown
333.1165	--	--	No MS-MS fragments obtained	--	unknown
377.1428	--	C10H26N4O9 P	No MS-MS fragments obtained	--	unknown

160.09	$[M+H]^+$	C7H13NO3	142.0860, 100.0760		Dehydrocarnitine
421.1682	$[M+H]^+$	C14H25N6O9<br>C7H13N3O5	363.1629, 305.1567	25	unknown
416.2134	--	C17H29N5O7	No MS-MS fragments obtained		unknown
289.0901	--	--	No MS-MS fragments obtained		unknown
170.0931	$[M+H]^+$	C7H12N3O2	124.0852, 109.0742, 97.1008, 96.0728, 95.0658, 83.0575	20	3-methylhistidine
137.0718	$[M+H]^+$	C7 H9 N2 O	120.0463, 110.0600, 94.0656	20	6-methylpyridine-3-carboxamide
207.0592	$[M+H]^+$	C4H9N5O5	165.0481, 132.0680	20	unknown

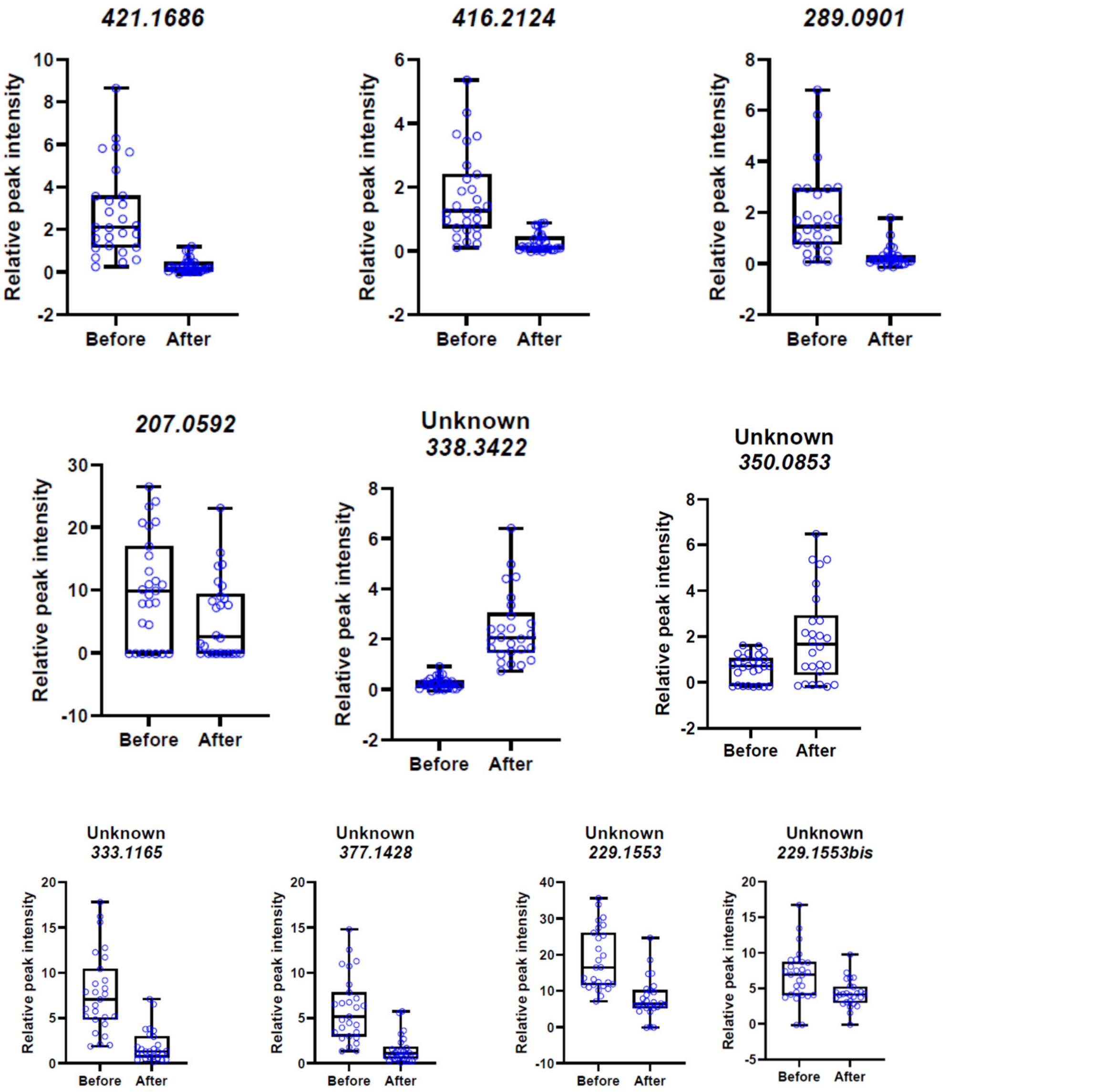

Figure S6. Boxplot of signal intensities of non-identified metabolites before and 9 h after meal intake. Legend: group 1 = before meal intake, group 2 = 9h after meal intake.
